# COMMUNITY BELONGING THROUGH THEATER: CREATIVE ARTS INTERVENTION FOR LOW-INCOME OLDER ADULTS

**DOI:** 10.1093/geroni/igz038.714

**Published:** 2019-11-08

**Authors:** Ruth E Dunkle, Laura Sutherland, Garrett T Pace, Ariel Kennedy, Patricia Baldwin

**Affiliations:** 1 University of Michigan, Ann Arbor, Michigan, United States; 2 Wayne State University, Detroit, Michigan, United States; 3 Hannan Foundation, Detroit, Michigan, United States

## Abstract

Creative arts can promote social contact and possibly reduce isolation. A professionally run theater group comprised of low-income older adults met for 12 weeks to learn basic skills and perform a play. Using a pre-post questionnaire, data were gathered from the treatment group (n=14) who participated in the class and a non-participating comparison group (n=5) to identify potential program effects on measures of social isolation, community belonging, and social exclusion. Participants were African American living in low-income housing in an urban area. The average age of the sample was 65 years, 21% were men, 83% had at least high school degree, 71% reported good to excellent health, and 58% reported at least one ADL. Regression analyses showed that a sense of community belonging was significantly greater for the treatment group than the comparison group at time 2.This was not the case when considering social isolation or social exclusion. When controls were added (age, health, and previous theater experience), the significant difference remained with higher age predicting a sense of community belonging. The greater number of class sessions attended was also associated with a greater sense of community belonging for the treatment group. Through the shared experience of theater, participants can gain a sense of community, but this activity does not seem to be related to social isolation or social exclusion. It could be that theater participation fosters a sense of belonging due to group dynamics but is not a significant enough activity to reduce a sense of isolation or exclusion.

